# Prognostic Value of HMGA2 in Human Cancers: A Meta-Analysis Based on Literatures and TCGA Datasets

**DOI:** 10.3389/fphys.2018.00776

**Published:** 2018-06-26

**Authors:** Ben Huang, Jiayi Yang, Qingyuan Cheng, Peipei Xu, June Wang, Zheng Zhang, Wei Fan, Ping Wang, Mingxia Yu

**Affiliations:** ^1^Department of Clinical Laboratory, Zhongnan Hospital of Wuhan University, Wuhan, China; ^2^Hubei Provincial Shuiguohu High School, Wuhan, China; ^3^Department of Pathology, Zhongnan Hospital of Wuhan University, Wuhan, China

**Keywords:** HMGA2, cancer, prognosis, TCGA, meta-analysis

## Abstract

**Background:** Emerging evidences have shown that the high-mobility group protein A2 (HMGA2) can aberrantly express in human cancers, and it could be an unfavorable prognostic factor in cancer patients. However, the prognostic value of HMGA2 was still unclear. Therefore, in this study, we explored the potential prognostic value of HMGA2 in human cancers by using meta-analysis based on published literatures and The Cancer Genome Atlas (TCGA) datasets.

**Methods:** Through searching PubMed, Embase, Web of Science and Cochrane Library databases, we were able to identify the studies evaluating the prognostic value of HMGA2 in cancers. Then, UALCAN and TCGA datasets were used to validate the results of our meta-analysis.

**Results:** In all, 15 types of cancers were included in this meta-analysis. Pooled results showed that high level of HMGA2 was significantly correlated with poor OS (HR = 1.88, 95% confidence interval (CI) = 1.68-2.11, *P* < 0.001) and poor DFS (HR = 2.49, 95% CI = 1.44-4.28, *P* = 0.001) in cancer patients. However, subgroup analyses revealed that the high expressed HMGA2 was associated with poor OS in head and neck cancer, gastric cancer and colorectal cancer, but not esophageal cancer and ovarian cancer. Based on TCGA datasets, we analyzed 9944 patients with 33 types of cancers. Significant association between HMGA2 overexpression and poor OS was found in 14 types of cancers. Taken together, consistent results were observed in clear cell renal cell carcinoma, esophageal adenocarcinoma, head and neck cancer, hepatocellular carcinoma, ovarian carcinoma, and pancreatic ductal adenocarcinoma.

**Conclusion:** Our meta-analysis showed the significance of HMGA2 and its prognostic value in various cancers. High level of HMGA2 could be associated with poor OS in patients with clear cell renal cell carcinoma, head and neck cancer, hepatocellular carcinoma and pancreatic ductal adenocarcinoma, but not esophageal adenocarcinoma and ovarian carcinoma.

## Introduction

Cancer has been one of the major causes of death and threats to global health due to its high morbidity and mortality (Wu S. et al., 2016). Meanwhile, the global incidence of cancer is rapidly increasing (Torre et al., 2015). Study shows that in 2017 there are 1,688,780 new cancer cases and 600,920 cancer deaths in the United States (Siegel et al., 2017). Many researchers have been focusing on identifying new tumor biomarkers which can be associated with cancer screening, diagnosis, prognosis, and evaluation of treatment efficacy (Ribeiro et al., 2016; Gorin et al., 2017; Kojima et al., 2017). Currently, even though various therapeutic methods have made significant achievements in cancer therapy, the 5-year-survival rate still remains unsatisfactory in cancer patients (Gonzalez and Agudo, 2012). Therefore, it is necessary to identify new prognostic biomarkers for accurate prognosis prediction.

High mobility group protein A2 (HMGA2) is a small non-histone chromosomal protein. It has no intrinsic transcriptional activity, but can modulate transcription by altering chromatin architecture (Hock et al., 2007; Pallante et al., 2015). Normally, HMGA2 protein is highly expressed in embryogenesis, while its expression is almost undetectable in most adult and differentiated tissues (Chiappetta et al., 1995; Rogalla et al., 1996; Hirning-Folz et al., 1998). An increasing number of recent studies have suggested that HMGA2 could highly express in many human malignant cells, and it can participate in different cellular activities including cell cycle regulation, differentiation and senescence (Wood et al., 2000; Hristov et al., 2009). HMGA2 overexpression has been reported to be associated with tumorigenesis and progression in many human cancers such as esophageal squamous cell cancer (Zhang et al., 2016), lung cancer (Kumar et al., 2014), pancreatic cancer (Piscuoglio et al., 2012), breast cancer (Wu J. et al., 2016) and colorectal cancer (Wang et al., 2011). Meanwhile, plenty of studies showed that elevated HMGA2 expression in cancer tissues was associated with poor survival in patients such as lung cancer (Sarhadi et al., 2006), oral squamous cell carcinoma (Miyazawa et al., 2004), ovarian cancer (Shell et al., 2007) and metastatic breast cancer (Langelotz et al., 2003). However, due to the limitations of sample size and research programs, single studies are inadequate to obtain a reliable assessment of the potential prognostic value of HMGA2. Therefore, we conducted a meta-analysis of a large sample size to gain better insight into this problem. In addition, The Cancer Genome Atlas (TCGA) databases were utilized to validate the result of our meta-analysis.

## Materials and Methods

### Study Strategy

Method of the analysis was based on the Preferred Reporting Items for Systematic Reviews and Meta-Analyses (PRISMA) statement (Moher et al., 2009). We searched PubMed, Embase, Web of Scienceand Cochrane Library databases for relevant articles from January 1, 2000 to August 17, 2017. The research subject and article language were limited to human and English. Both MeSH terms and free-text words were used as strategy to increase the sensitivity of the searching. The search terms included: (“cancer” OR “tumor” OR “neoplasm” OR “carcinoma”) AND (“High mobility group protein A2” OR “HMGA2”) AND (“prognosis” OR “prognostic” OR “outcome”). We also manually screened the references of retrieved articles to identify more eligible studies that may have been missed by the key word search.

### Inclusion and Exclusion Criteria

Two authors (Ben Huang and Jiayi Yang) independently selected all eligible articles according to the criteria: (1) studies that showed the association between HMGA2 expression and prognosis of cancers patients as well as reporting survival data; (2) studies that provided related clinicopathological parameters; (3) studies in which number of patients were more than 40.

Exclusion criteria were as followed: (1) articles that lack data; (2) reviews, letters, case reports or duplicates.

### Data Extraction and Quality Assessment

Two investigators independently extracted the relevant data from the included articles using a predefined standardized form. We extracted the following data from each study: (1) publication year; (2) countries; (3) first author’s name; (4) types of cancers; (5) detection methods; (6) number of patients; (7) cut off values; (8) clinicopathological parameters; (9) relevant data for OS/PFS/DFS. If the articles did not offer HRs and its 95% CIs directly, we then used the Engauge Digitizer 4.1 software (Tierney et al., 2007) to extract the data from the survival curves. Disagreements in the literature assessment were resolved through consensus with a third reviewer (June Wang). Furthermore, we used the Newcastle-Ottawa Scale (NOS) to assess the quality of these included studies (Zeng et al., 2015). According to the NOS criteria, studies with a score of ≥7 were considered to be high quality articles.

### Extraction and Analysis of TCGA Datasets

Datas for HMGA2 expression and clinical information in TCGA were extracted from the UALCAN^1^ (Chandrashekar et al., 2017). In all, 33 types of cancers were analyzed. There were 9944 subjects that have both HMGA2 expression data and survival data. Accorrding to the transcripts per million (TPM) expression value, the HMGA2 expression levels were divided into high expression group (with TPM values above upper quartile) and low/median expression group (with TPM values below upper quartile). Then, according to the survival data of patients in TCGA datasets, Kaplan*–*Meier survival analyses were performed and overall survival plots were generated. The difference between high gene expression and low/medium gene expression was compared by Log-rank test.

### Outcomes Analysis

Pooled HRs and corresponding 95% CIs were calculated to evaluate the impact of HMGA2 expression on overall survival and disease-free survival. ORs with corresponding 95% CIs were calculated to assess the correlation between HMGA2 expression and clinicopathological features. Chi square-based *Q* test and *I*^2^ test were used to evaluate the heterogeneity across the studies. A fixed-effect model was adopted if the heterogeneity was not significant (*I*^2^ < 50% or *P*-value > 0.05). Otherwise, a random-effect model was selected. Besides, subgroup analysis was performed to explore the sources of heterogeneity. Meanwhile, we used Stata12.0 software (Stata Corporation, College Station, TX, United States) to analyze the sensitivity and publication bias in this study. All the statistical tests were two-sided, and *P* < 0.05 was considered statistically significant.

## Results

### The Description of the Included Studies

**FIGURE 1:**
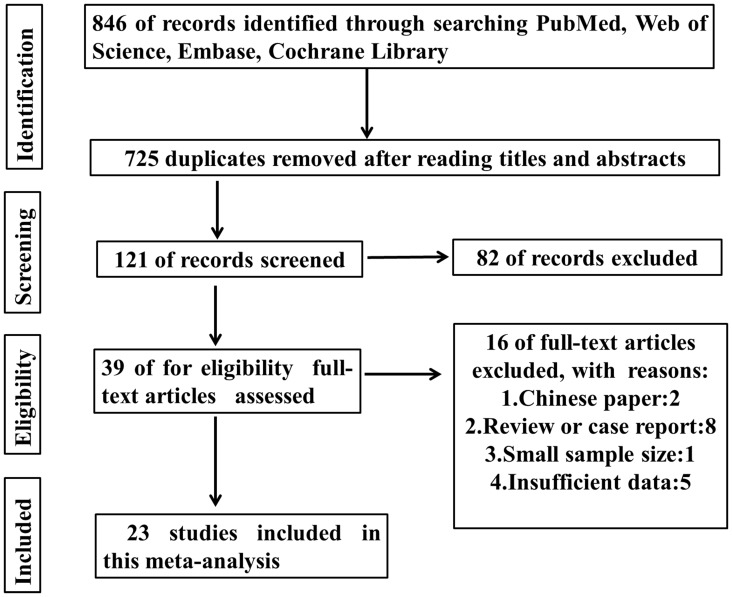
Flow diagram of study selection.

As shown in the **Figure 1**, 846 records were obtained by searching the databases. After screening the titles and abstracts, 807 articles were excluded. Then 16 papers were excluded because of no available data or non English papers. Eventually, 23 articles were enrolled (Motoyama et al., 2008; Wang et al., 2011; Yang et al., 2011; Wu et al., 2012; Rizzi et al., 2013; Yamazaki et al., 2013; Califano et al., 2014; Kong et al., 2014; Lee et al., 2014, 2015; Jun et al., 2015; Kim et al., 2015; Liu et al., 2015; Xia et al., 2015b; Na et al., 2016; Wei et al., 2016; Wu J. et al., 2016; Zhang et al., 2016; Zhao et al., 2016; Fang et al., 2017; Gunther et al., 2017; Mito et al., 2017; Strell et al., 2017). In total, 15 types of cancers were included in this meta-analysis including ampullary adenocarcinoma, breast cancer, colorectal cancer, clear cell renal cell carcinoma (ccRCC), esophageal adenocarcinoma, esophageal squamous cell carcinoma, gastric cancer, head and neck squamous cell carcinoma, hepatocellular carcinoma, intrahepatic cholangiocarcinomas, nasopharyngeal carcinoma, ovarian carcinoma, oral squamous cell carcinoma, pancreatic ductal adenocarcinoma, and tongue squamous cell carcinoma. In these studies, the level of HMGA2 expression was all detected in collected tumor tissues. Almost all the studies performed immunohistochemistry (IHC) to evaluate the expression of HMGA2 while only one of them used the relative quantitative reverse transcription-polymerase chain reaction (qT-PCR). The main features of the eligible studies were listed in **Table 1**.

**Table 1:** Characteristics of eligible studies in this meta-analysis.

First author	Year	Country	No. of patients	Tumor type	Method	Cut-off	Outcome	Analysis	Nos
Strell	2017	Sweden	253	Pancreatic ductal denocarcinoma	ICH	IHC score ≥ 1	OS	Multivariate	7
Strell	2017	Sweden	155	Ampullary adenocarcinoma	ICH	IHC score ≥ 1	OS	Multivariate	7
Mito	2017	USA	91	Esophageal Adenocarcinoma	ICH	NR	OS	Multivariate	8
Fang	2017	China	148	Oral Squamous Cell Carcinoma	ICH	IHC ≥ 30%	OS/DFS	Multivariate	9
Wei	2017	China	96	Esophageal squamous cell carcinoma	ICH	IHC score ≥ 4	OS	Multivariate	8
Gunther	2016	Germany	202	Head and neck squamous cell carcinoma	RT-PCR	upper quartile	OS/PFS	Multivariate	8
Wu	2016	China	273	Breast cancer	ICH	NR	OS	Multivariate	7
Zhao	2016	China	60	Tongue squamous cell carcinoma	ICH	NR	OS/DFS	Multivariate	9
Na	2016	China	162	Clear cell renal cell carcinoma	ICH	IHC score > 106	OS	Multivariate	9
Zhang	2016	China	127	Esophageal squamous cell carcinoma	ICH	IHC score ≥ 4	OS	Multivariate	8
Jun	2015	Korea	110	Gastric cancer	ICH	IHC score ≥ 5	RFS	Multivariate	9
Kim	2015	Korea	74	Ovarian carcinoma	ICH	NR	OS	Multivariate	9
Liu	2015	China	116	Nasopharyngeal carcinoma	ICH	IHC score ≥ 4	OS	Multivariate	8
Xia	2015	China	124	Nasopharyngeal carcinoma	ICH	IHC score ≥ 6	OS	Multivariate	8
Lee	2015	Korea	170	Gastric cancer	ICH	IHC score ≥ 9	OS	Multivariate	9
Kong	2014	China	158	Gastric cancer	ICH	IHC score ≥ 1	OS	Multivariate	8
Lee	2014	China	55	Intrahepatic cholangiocarcinomas	ICH	IHC > 5%	OS	Multivariate	7
Rizzi	2013	Italy	103	Colorectal cancer	ICH	IHC ≥ 5%	OS	Kaplan-Meier curves	7
Califano	2013	Italy	113	Ovarian cancer	ICH	IHC ≥ 10%	OS/DFS	Multivariate	7
Yamazaki	2013	Japan	91	Head and neck squamous cell carcinoma	ICH	IHC score ≥ 1	OS	Kaplan-Meier curves	8
Wu	2012	China	107	Hepatocellular Carcinoma	ICH	IHC ≥ 10%	OS	Multivariate	8
Yang	2011	China	148	Bladder cancer	ICH	IHC ≥ 50%	PFS/RFS	Multivariate	9
Wang	2011	China	89	Colorectal Cancer	ICH	IHC ≥ 5%	OS	Multivariate	8
Wang	2011	China	191	Colorectal Cancer	ICH	IHC ≥ 5%	OS	Multivariate	8
Motoyama	2008	Japan	110	Gastric cancer	ICH	NR	OS	Multivariate	8

*ICH, Immunohistochemistry; NR, Not Reported; NOS, Newcastle-Ottawa Scale; OS, overall survival; DFS, disease-free survival*.

### Meta-Analysis of HMGA2 Expression on OS/DFS

**FIGURE 2:**
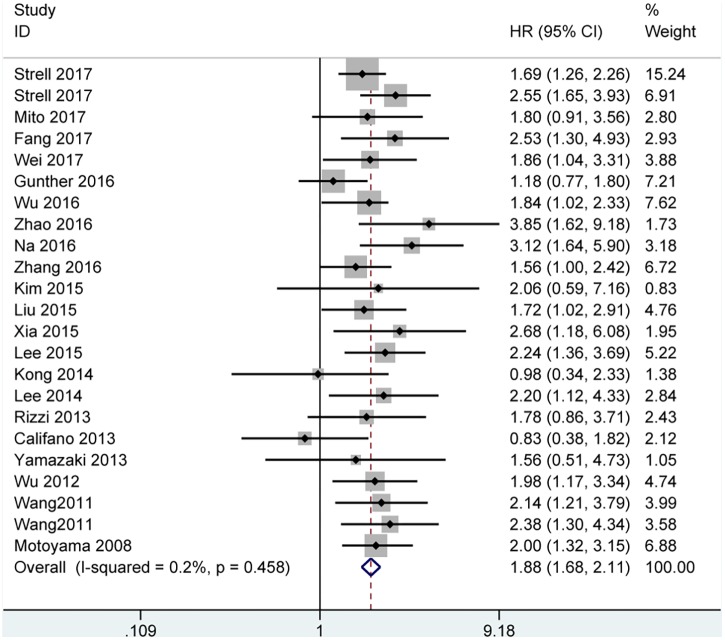
Forest plot of studies evaluating hazard ratios of high expressed HMGA2 and the overall survival of cancer patients.

**FIGURE 3:**
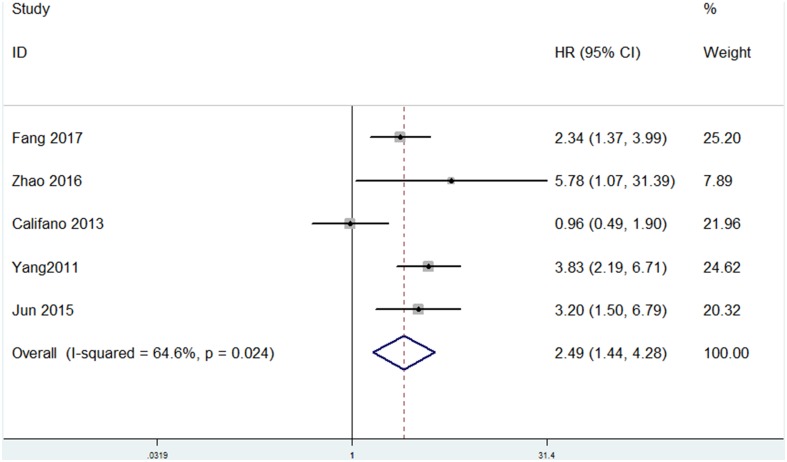
Forest plot of HR for the correlation between HMGA2 expression and Disease-free survival (DFS) in cancers.

Among the included papers, 23 studies involving 3068 patients showed the data of both HMGA2 expression and OS of the patients. As displayed in **Figure 2**, there was no obvious heterogeneity across these studies (*I*^2^ = 0.2%, *P* = 0.458). Thus, we used the fixed-effect model to evaluate the pooled HRs and 95% CIs. As a result, the pooled data indicated that elevated HMGA2 was significantly associated with poor OS in patients with cancers (HR = 1.88, 95% CI = 1.68-2.11, *P* < 0.001). Meanwhile, there were five studies showed the association between HMGA2 expression level and DFS in the included studies. Heterogeneity test indicated there was an obvious heterogeneity (*I*^2^ = 64.6%), then the random-effect model was used. Pooled results also demonstrated that high HMGA2 expression was associated with shorter DFS in cancer patients (HR = 2.49, 95% CI = 1.44-4.28) (**Figure 3**).

### Subgroup Analysis for OS

We then made a subgroup analysis for OS, in which the patients were stratified based on cancer type, analysis type and sample size (**Table 2**). There was only one study for each that evaluated the association between HMGA2 expression and OS in pancreatic ductal adenocarcinoma, ampullary adenocarcinoma, breast cancer, ccRCC, intrahepatic cholangiocarcinoma, and hepatocellular carcinoma. Therefore, we defined these cancers as “other cancers” in this subgroup. Results show that high expression level of HMGA2 was associated with poor OS in gastric cancer (HR = 1.94, 95% CI = 1.42-2.65, *P* < 0.001), head and neck cancer (HR = 1.77, 95% CI = 1.37-2.29, *P* < 0.001), colorectal cancer (HR = 2.13, 95% CI = 1.48-3.05, *P* < 0.001) and other cancers (HR = 2, 95% CI = 1.68-2.40, *P* < 0.001), but not esophageal cancer (HR = 1.15, 95% CI = 0.55-2.37, *P* = 0.712) and ovarian cancer (HR = 1.07, 95% CI = 0.55-2.37, *P* = 0.712). As a result, we found that high level of HMGA2 was related with poor OS in 13 types of cancers. Meanwhile, in the subgroups based on sample size and analysis type, we also found the association between HMGA2 and poor OS except for multivariate analysis.

**Table 2:** Subgroup analyses of pooled HR for OS.

Categories	No. of studies	No. of patients	Pooled HR (95% CI)		Heterogeneity	
			Fix/Random	*p*-value	*I*^2^(%)	*P*-value
**[1] OS**	23	3068	1.88 (1.68-2.11)	0	0.2	0.458
**[2] Cancer type**
1) Colorectal cancer	3	383	2.13(1.48-3.05)	0	0	0.834
2) Esophageal cancer	3	314	1.15 (0.55-2.37)	0.712	80	0.007
3) Gastric cancer	3	438	1.94 (1.42-2.65)	0	11.4	0.324
5) Head and neck cancer	6	741	1.77 (1.37-2.29)	0	42.8	0.12
6) Ovarian cancer	2	187	1.07 (0.55-2.08)	0.835	31.6	0.227
7) Others	8	1213	2.09 (1.76-2.47)	0	0	0.43
**[3] Analysis**
Multivatiate	21	2874	1.80 (1.60-2.02)	0	48.9	0.006
Survival curves	2	194	1.71 (0.93-3.15)	0.085	0	0.864
**[4] Sample size**
≥115	12	2079	1.85 (1.61-2.13)	0	22	0.227
<115	11	989	1.68 (1.21-2.33)	0.002	59.1	0.006

*OS, overall survival; HR, hazard ratio*.

### HMGA2 Overexpression and Clinical Pathological Features

In order to gain further insight into the value of HMGA2, we investigated the association between HMGA2 level and certain clinicopathological parameters in cancers (**Table 3**). The expression level of HMGA2 was significantly associated with TNM stage (OR = 1.65, 95% CI = 1.12-2.44, *P* = 0.011, random-effect model), tumor differentiation (OR = 1.94, 95% CI = 1.51-2.51, *P* < 0.001, fix-effect model), tumor invasion depth (OR = 1.71, 95% CI = 1.35-3.16, *P* < 0.001, fix-effect model), lymph node metastasis (OR = 1.86, 95% CI = 1.27-2.72, *P* = 0.001, random-effect model), lymphovascular invasion (OR = 2.18, 95% CI = 1.49-3.18, *P* < 0.001, fix-effect model), vascular invasion (OR = 2.1, 95% CI = 1.42-3.10, *P* < 0.001, fix-effect model) and distant metastasis (OR = 3.45, 95% CI = 2.06-5.75, *P* < 0.001, fix-effect model). No significant correlations were found with age (OR = 0.99, 95% CI = 0.74-1.32, *P* = 0.931, fixed-effect model), gender (OR = 1, 95% CI = 0.84-1.18, *P* = 0.974, fixed-effect model) and tumor size (OR = 0.77, 95% CI = 0.53-1.14, *P* = 0.19, fixed-effect model). The results indicated that high HMGA2 expression in human cancers was linked to aggressive biological behavior.

**Table 3:** Clinicopathological features of the enrolled studies with high expressed HMGA2 in patients with cancer.

Clinicopathological parameters	Studies	No. of patients	Risk of high HMGA2 OR (95% CI)	Significant *Z*	*p*-value	Heterogeneity *I*^2^ (%)	*p*-value	Model
Age (<50 vs ≥50)	6	919	0.99 (0.74-1.32)	0.09	0.931	0	0.836	Fixed effects
Gender (male vs female)	21	2803	1.00 (0.84-1.18)	0.03	0.974	27.3	0.122	Fixed effects
Tumor size (<3 cm vs ≥3 cm)	4	543	0.77 (0.53-1.14)	1.31	0.19	0	0.756	Fixed effects
TNM stage (III-IV vs I-II)	16	2292	1.65 (1.12-2.44)	2.53	0.011	68.4	0	Random effects
Tumor differentiation (moderate/well vs poor)	10	1317	1.94 (1.51-2.51)	5.11	0	46.9	0.049	Fixed effects
Tumor invasion depth (T3–T4 vs T1–T2)	13	1767	1.71 (1.35-3.16)	4.52	0	40.7	0.063	Fixed effects
Distant metastasis (Positive vs negative)	6	721	3.45 (2.06-5.75)	4.73	0	41.7	0.127	Fixed effects
Lymph node metastasis (Positive vs negative)	17	2289	1.86 (1.27-2.72)	3.19	0.001	74.9	0	Random effects
Lymphovascular invasion (Positive vs negative)	5	629	2.18 (1.49-3.18)	4.02	0	31.4	0.212	Fixed effects
Vascular invasion (Positive vs negative)	5	655	2.1 (1.42-3.10)	3.73	0	0	0.464	Fixed Effects

### Sensitivity Analyses and Publication Bias

**FIGURE 4:**
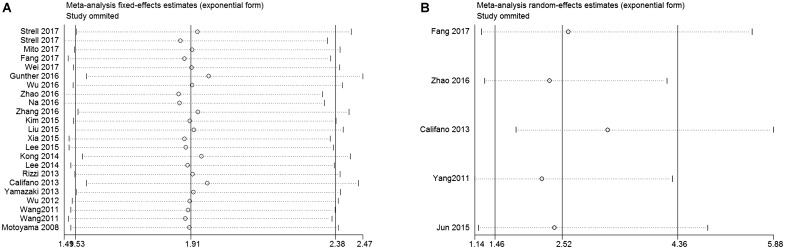
Sensitivity analysis of this meta-analysis. **(A)** Overall survival (OS). **(B)** Disease-free survival (DFS).

**FIGURE 5:**
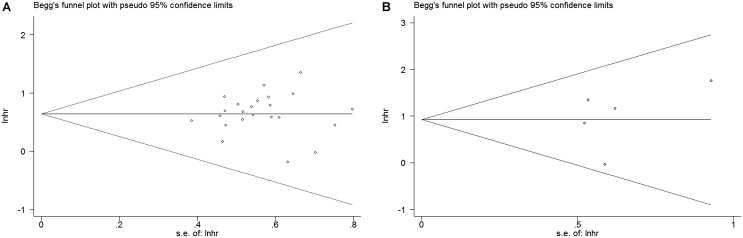
Begg’s funnel plots for the studies involved in the meta-analysis of HMGA2 expression and the prognosis of patients with cancers. **(A)** Overall survival. **(B)** Disease-free survival. loghr, logarithm of hazard ratios; s.e., standard error.

In order to assess whether a single study could significantly affect the overall result, we performed sensitivity analyses. Results demonstrated the individual study had no influence on our meta-analysis (**Figure 4**), which supported credibility of our analysis. In addition, Funnel plots and Begg’s test were used to evaluate the publication bias of this meta-analysis. Results showed that there was no publication bias existed in studies on HMGA2 overexpression in association with OS (*P* = 0.597. **Figure 5A**) and DFS (*P* = 0.462. **Figure 5B**).

### The Expression of HMGA2 in Cancers Based on TCGA Datasets

We then analyzed the expression of HMGA2 in different cancers in TCGA datasets through UALCAN, which was an interactive web-portal to perform in-depth analyses of TCGA gene expression data (Chandrashekar et al., 2017). HMGA2 was detected in 21 types of cancers and corresponding normal tissues. According to *P* value obtained from log-rank test, we found that the HMGA2 expression was significantly higher than corresponding normal tissues in 17 types of cancers except glioblastoma multiforme, kidney chromophobe, kidney renal clear cell carcinoma and prostate adenocarcinoma (**Table 4**).

**Table 4:** The difference of HMGA2 expression in cancers and corresponding normal tissues in TCGA datasets.

Types of cancer	TCGA dataset	No. of cancer tissues	No. of normal tissues	*P* value
Bladder urothelial carcinoma	TCGA-BLCA	408	19	<0.0001
Breast invasive carcinoma	TCGA-BRCA	1097	114	<0.0001
Cervical squamous cell carcinoma	TCGA-CESC	305	3	<0.0001
Cholangiocarcinoma	TCGA-CHOL	36	9	0.032
Colon adenocarcinoma	TCGA-COAD	286	41	<0.0001
Esophageal carcinoma	TCGA-ESCA	184	11	<0.0001
Glioblastoma multiforme	TCGA-GBM	156	5	0.052
Head and Neck squamous cell carcinoma	TCGA-HNSC	520	44	<0.0001
Kidney chromophobe	TCGA-KICH	67	25	0.316
Kidney renal clear cell carcinoma	TCGA-KIRC	533	72	0.071
Kidney renal papillary cell carcinoma	TCGA-KIRP	290	32	<0.0001
Liver hepatocellular carcinoma	TCGA-LIHC	371	50	<0.0001
Lung adenocarcinoma	TCGA-LUAD	515	59	<0.0001
Lung squamous cell carcinoma	TCGA-LUSC	503	52	<0.0001
Ovarian serous cystadenocarcinoma	TCGA-OV	178	4	<0.0001
Pheochromocytoma and Paraganglioma	TCGA-PCPG	179	3	0.011
Prostate adenocarcinoma	TCGA-PRAD	497	52	0.201
Rectum adenocarcinoma	TCGA-READ	166	10	<0.0001
Stomach adenocarcinoma	TCGA-STAD	415	34	<0.0001
Thyroid carcinoma	TCGA-THCA	505	59	<0.0001
Uterine corpus endometrial carcinoma	TCGA-UCEC	546	35	<0.0001

### Validation by TCGA Datasets

**FIGURE 6:**
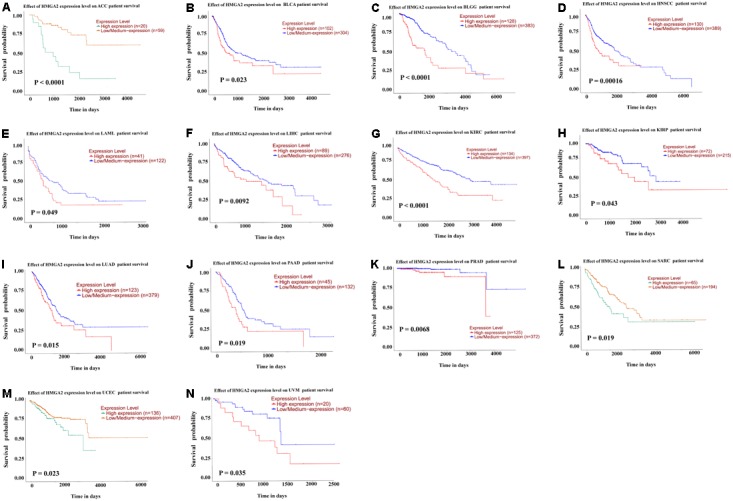
Kaplan–Meier survival curves for cancer patients based on TCGA datasets. **(A)** adrenocortical carcinoma. **(B)** bladder urothelial carcinoma. **(C)** brain lower grade glioma. **(D)** head and neck squamous cell carcinoma. **(E)** kidney renal clear cell carcinoma. **(F)** kidney renal papillary cell carcinoma. **(G)** acute myeloid leukemia. **(H)** liver hepatocellular carcinoma. **(I)** lung adenocarcinoma. **(J)** pancreatic adenocarcinoma. **(K)** prostate adenocarcinoma. **(L)** sarcoma. **(M)** uterine corpus endometrial carcinoma. **(N)** uveal melanoma.

In order to validate the result of our meta-analysis, TCGA datasets were analyzed to explore whether HMGA2 could be involved in human cancers and affect patients’ survival. In total, 9944 patients with 33 types of cancer were obtained (**Table 5**). Significant association between high expressed HMGA2 and poor overall survival in patients was found in 14 types of cancers. They were adrenocortical carcinoma, bladder urothelial carcinoma, brain lower grade glioma, head and neck squamous cell carcinoma, kidney renal clear cell carcinoma, kidney renal papillary cell carcinoma, acute myeloid leukemia, liver hepatocellular carcinoma, lung adenocarcinoma, pancreatic adenocarcinoma, prostate adenocarcinoma, sarcoma, uterine corpus endometrial carcinoma and uveal melanoma (**Figure 6**).

**Table 5:** The difference of overall survival in cancer patients with high HMGA2 expression vs low/median expression.

Cancer type	No. of cancer tissues			*P* value
	Total	High	Low/Median	
ACC	79	20	59	<0.0001
BLCA	406	102	304	0.023
BLGG	511	128	383	<0.0001
BRCA	1081	273	808	0.72
CESC	291	77	214	0.3
CHOL	36	9	27	0.14
COAD	279	67	212	0.38
ESCA	184	46	138	0.76
GBM	152	39	113	0.13
HNSCC	519	130	389	0.00016
KICH	65	17	48	0.39
KIRC	531	134	397	<0.0001
KIRP	287	72	215	0.043
LAML	163	41	122	0.049
LIHC	365	89	276	0.0092
LUAD	502	123	379	0.015
LUSC	494	123	371	0.28
DLBC	47	11	36	0.21
MESO	85	22	63	0.56
OVSC	303	76	227	0.83
PAAD	177	45	132	0.019
PCPG	179	45	134	0.23
PRAD	497	125	372	0.0068
READ	165	41	124	0.52
SARC	259	65	194	0.019
SKCM	459	115	344	0.61
STAD	392	98	294	0.49
TGCT	134	30	104	0.26
THYM	119	30	89	0.052
THCA	504	127	377	0.64
UCS	56	15	41	0.57
UCEC	543	136	407	0.023
UVM	80	20	60	0.035

*ACC, adrenocortical carcinoma; BLCA, bladder urothelial carcinoma; BLGG, brain lower grade glioma; BRCA, breast invasive carcinoma; CESC, cervical squamous cell carcinoma; CHOL, cholangiocarcinoma; COAD, colon adenocarcinoma; ESCA, esophageal carcinoma; GBM, glioblastoma multiforme; HNSCC, head and neck squamous cell carcinoma; KICH, kidney chromophobe; KIRC, kidney renal clear cell carcinoma; LAML, acute myeloid leukemia; LIHC, liver hepatocellular carcinoma; LUAD, lung adenocarcinoma; LUSC, lung squamous cell carcinoma; DLBC, lymphoid neoplasm diffuse large B-cell lymphoma; MESO, mesothelioma; OVSC, ovarian serous cystadenocarcinoma; PAAD, pancreatic adenocarcinoma; PCPG, pheochromocytoma and paraganglioma; PRAD, prostate adenocarcinoma; READ, rectum adenocarcinoma; SARC, sarcoma; SKCM, skin cutaneous melanoma; STAD, stomach adenocarcinoma; TGCT, testicular germ cell tumors; THYM, thymoma; THCA, thyroid carcinoma; UCS, uterine carcinosarcoma; UCEC, uterine corpus endometrial carcinoma; UVM, uveal melanoma*.

Considering the TCGA datasets and our meta-analysis, we identified correlation between high HMGA2 expression and head and neck cancer, pancreatic ductal adenocarcinoma, ccRCC, hepatocellular carcinoma, esophageal adenocarcinoma and ovarian carcinoma, except breast cancer and gastric cancer.

## Discussion

Recently, increasing evidences have suggested that HMGA2 protein could participate in aggressive tumor growth (Peng et al., 2008; Hristov et al., 2009), stem cell self-renewal (Nishino et al., 2008; Tzatsos and Bardeesy, 2008), DNA damage response (Li et al., 2009), and tumor cell differentiation (Shell et al., 2007). However, the precise role of HMGA2 in malignant transformation and the gene regulation of tumorigenesis were still unclear (Venkatesan et al., 2012). Data have collectively indicated that the high level of HMGA2 could serve as a novel biomarker to evaluate the prognosis of patients with various cancers such as breast cancer (Wu J. et al., 2016), lung cancer (Di Cello et al., 2008), ovarian cancer (Mahajan et al., 2010), colorectal cancer (Wang et al., 2011), and gastric cancer (Lee et al., 2015). However, single studies may not be sufficient and accurate and whether HMGA2 could be used as a prognostic biomarker in human cancers was still unclear.

To the best of our knowledge, our meta-analysis was the first study to evaluate the significance of HMGA2 and prognostic value in various cancers through drawing data from both the TCGA datasets and published studies. In this paper, 15 types of cancers involving 3068 patients were included. Then, based on TCGA datasets, we analyzed 9944 patients with 33 types of cancers. The meta-analysis results suggested that high expression of HMGA2 was associated with shorter OS and DFS in patients with cancers. However, in the subgroup analysis for OS, we only found the association in 13 types of cancers except for the ones in esophageal adenocarcinoma and ovarian carcinoma. In addition, through TCGA datasets, we observed the poor prognosis in 14 types of cancers. Consistent results were found in six types of cancers. Patients with high expressed HMGA2 in ccRCC, head and neck cancer, hepatocellular carcinoma and pancreatic ductal adenocarcinoma showed a significant shorter OS than patients with a low level of HMGA2 expression. However, in esophageal adenocarcinoma and ovarian carcinoma, we did not observe any significant correlation.

We then explored the relationship between clinicopathological features and high HMGA2 expression in our enrolled studies. We found the level of HMGA2 was positively associated with TNM stage, tumor differentiation, tumor invasion depth, lymph node metastasis, lymphovascular invasion, and vascular invasion, which indicated that HMGA2 might have a significant relationship with advanced features of cancer. Previous research performed by Di Cello et al. (2008) found that HMGA2 protein levels were increased in all metastatic lung cancer cell lines compared with benign tumors and normal cells. According to Piscuoglio et al. (2012), HMGA2 might play a significant role in the late stages of pancreatic carcinogenesis and in the progression towards a more aggressive tumor phenotype. In addition, HMGA2 was demonstrated to play a critical role in epithelial-to-mesenchymal transition (EMT) in various cancers such as gastric cancer (Zha et al., 2013), hepatocellular carcinoma (Luo et al., 2013) and nasopharyngeal cancer (Xia et al., 2015a), thus inducing epithelial cancer invasion and metastasis.

Since the high expression of HMGA2 could be potentially an indicator of poor prognosis in patients with certain cancers, HMGA2 was expected to be a new therapeutic biomarker in cancers. Recently, Zhao et al. demonstrated that by directly targeting HMGA2, miR-599 could serve tumor suppressive roles in ccRCC (Zhao et al., 2018). A study conducted by Malek et al. (2008) suggested silencing HMGA2 expression in ovarian cancer cells could have a therapeutic effect on ovarian cancer. Liu et al. (2014) reported the raf kinase inhibitor protein can inhibit the survival and invasion of gastric cancer cells and promote apoptosis through regulating the expression of HMGA2 and Osteopontin. The findings of Chau et al. (2003) suggested that HMGA2 could become a therapeutic target by blocking HMGA2 protein expression in retinoblastoma cells or through inhibiting expression of the HMGA2 gene by targeting its promoters. Wang et al. (2011) found radiotherapy significantly reduced the relative risk death in HMGA2-positive colorectal cancers (CRCs), but not in HMGA2-negative CRCs. All these studies showed HMGA2 could play a key role in cancers, which supported it potential role as a biomarker for cancer therapy.

However, some limitations of this study should be acknowledged. Firstly, heterogeneity in our study was substantial, which might be attributed to differences in types of cancers, study areas and cut-off values. Secondly, in the process of data extraction, we evaluated the HRs and 95% CIs from the Kaplan–Meier survival curves in five studies rather than directly obtained from the studies, which might be less accurate than extracting directly from published statistics. Thirdly, since negative results would have little chance to be published, there may be a bias in the published studies. Therefore, although no significant publication bias was detected in this meta-analysis, the results of our meta-analysis still need to be verified by a larger number of studies.

Despite the limitations described, our meta-analysis revealed the significance of HMGA2. Our meta-analysis showed that HMGA2 likely played an important role in human cancers and overexpression of HMGA2 could be associated with aggressive biological behavior although its prognostic values varied in different types of cancers. Specifically, overexpression of HMGA2 was significantly associated with poor prognosis in patients with ccRCC, head and neck cancer, hepatocellular carcinoma and pancreatic ductal adenocarcinoma, but not esophageal adenocarcinoma and ovarian carcinoma.

## Author Contributions

BH, JY, and MY conceived the study. BH, JY, and JW searched the databases and extracted the data. WF, ZZ, and PX analyzed the data. BH wrote the draft of the paper. QC, PW, and MY reviewed the manuscript.

## Conflict of Interest Statement

The authors declare that the research was conducted in the absence of any commercial or financial relationships that could be construed as a potential conflict of interest.

## Footnotes

1 http://ualcan.path.uab.edu/index.html
